# Aortic perforation from central venous catheterization: a near-miss catastrophe

**DOI:** 10.1093/jscr/rjag521

**Published:** 2026-06-26

**Authors:** Kewei Jia, Xin Li, Bin Liao

**Affiliations:** Department of Cardiovascular Surgery, The Affiliated Hospital of Southwest Medical University, No. 8, Section 2, Kangcheng Road, Jiangyang District, Luzhou, Sichuan 646000, China; Department of Cardiovascular Surgery, The Affiliated Hospital of Southwest Medical University, No. 8, Section 2, Kangcheng Road, Jiangyang District, Luzhou, Sichuan 646000, China; Department of Cardiovascular Surgery, The Affiliated Hospital of Southwest Medical University, No. 8, Section 2, Kangcheng Road, Jiangyang District, Luzhou, Sichuan 646000, China

**Keywords:** aortic perforation, central venous catheterization, internal jugular vein catheterization, iatrogenic complication, vascular injury, emergency surgery

## Abstract

Central venous catheterization for hemodialysis access carries a risk of inadvertent arterial cannulation, which can lead to catastrophic complications, including aortic arch perforation. We report the case of a 61-year-old man with stage 5 chronic kidney disease who sustained an aortic perforation during an attempted right internal jugular vein cannulation at an outside institution. Computed tomography angiography revealed the catheter traversing the right common carotid artery and brachiocephalic trunk, with an extraluminal tip located in the aortic arch, accompanied by hemopericardium and a periaortic hematoma. Emergent surgical removal was performed under direct visualization with manual compression and standby cardiopulmonary bypass, and the patient recovered uneventfully. This case underscores the essential role of real-time ultrasound guidance in preventing arterial injury. When arterial cannulation is suspected, blind catheter withdrawal must be strictly avoided, and prompt imaging, immediate surgical consultation, and controlled catheter extraction are critical to averting fatal hemorrhage.

## Introduction

Central venous catheterization (CVC) for hemodialysis access is a common procedure, yet inadvertent arterial cannulation occurs, including aortic arch perforation, massive hemorrhage, and cardiac tamponade. Risk factors include patient anatomy and operator experience, with inadequate ultrasound guidance representing a key modifiable factor [1]. Real-time ultrasound guidance is the most effective preventive measure and is strongly recommended by international guidelines [2, 3]. When arterial cannulation is suspected, blind catheter withdrawal must be strictly avoided, as it may precipitate fatal hemorrhage [4]. Management principles derived from vascular trauma guidelines emphasize prompt imaging, multidisciplinary collaboration, and controlled surgical removal [5]. Aortic arch perforation from central venous access is exceedingly rare. We report the case of an aortic arch perforation following an attempted internal jugular vein cannulation in a 61-year-old man, highlighting the importance of early recognition and structured surgical management.

## Case presentation

A 61-year-old man with newly diagnosed stage 5 chronic kidney disease presented with an acute pulsatile hemorrhage from the right side of the neck immediately following an attempted internal jugular vein catheterization for hemodialysis access at an outside institution (Fig. 1, Supplementary Video S1). The patient had no prior history of cardiovascular disease or surgical intervention. On arrival, emergent contrast-enhanced computed tomography angiography (CTA) of the thoracic aorta and contrast-enhanced chest CT were performed. Imaging confirmed inadvertent arterial cannulation, with the catheter traversing the right common carotid artery and brachiocephalic trunk into the aortic arch, perforating the aortic wall, and terminating extraluminally (Figs 2 and 3). Contrast-enhanced chest CT additionally demonstrated hemopericardium and a periaortic hematoma. No aortic dissection or extension of mediastinal hematoma was observed. Given the high risk of fatal hemorrhage and cardiac tamponade with blind catheter withdrawal, emergency surgical exploration with cardiopulmonary bypass on standby was indicated.

**Figure 1:**
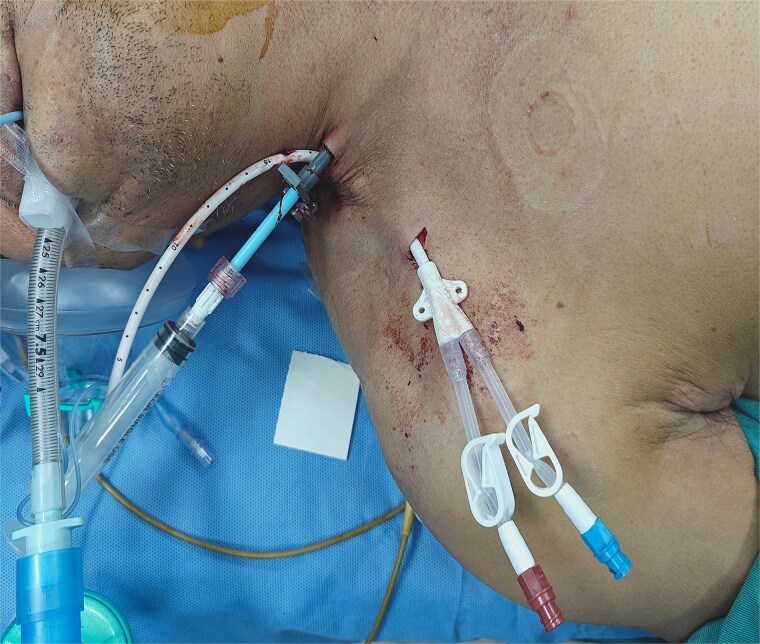
Preoperative photograph: the photograph shows the right side of the neck following internal jugular vein catheterization.

**Figure 2:**
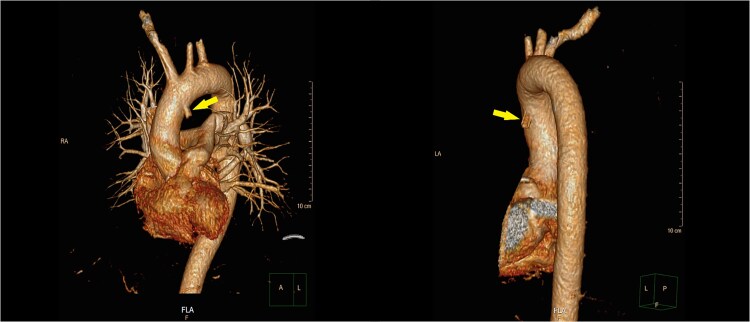
CTA images of the thoracic aorta: images show aortic arch perforation.

**Figure 3:**
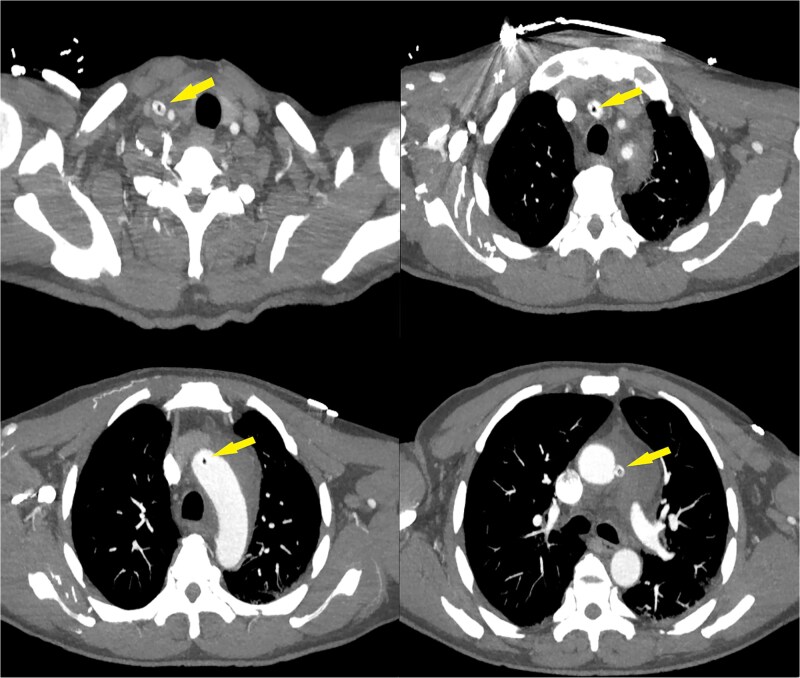
Contrast-enhanced chest CT images: images show the catheter traversing the right common carotid artery and brachiocephalic trunk into the aortic arch.

Emergency surgery was performed under general anesthesia. Intraoperative exploration revealed a hemopericardium and a periaortic hematoma measuring ~3 × 4 cm between the lesser curvature of the aortic arch and the main pulmonary artery trunk (Fig. 4). The catheter was observed entering the right common carotid artery, traversing the brachiocephalic trunk, and emerging at the proximal aortic arch. The ascending aorta was intact. After the evacuation of pericardial blood and clots (Supplementary Videos S2 and S3), manual compression was applied to the periaortic hematoma. The catheter and puncture needle were gradually withdrawn under direct visualization while sustained manual compression was maintained, and removal was accomplished without major bleeding (Fig. 5). No intraoperative complications occurred.

**Figure 4:**
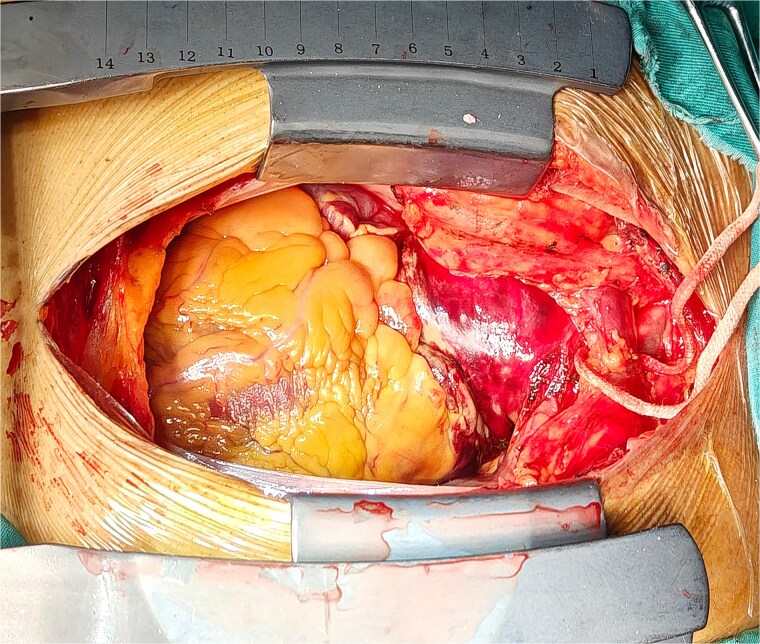
Intraoperative photograph: the photograph shows a periaortic hematoma.

**Figure 5:**
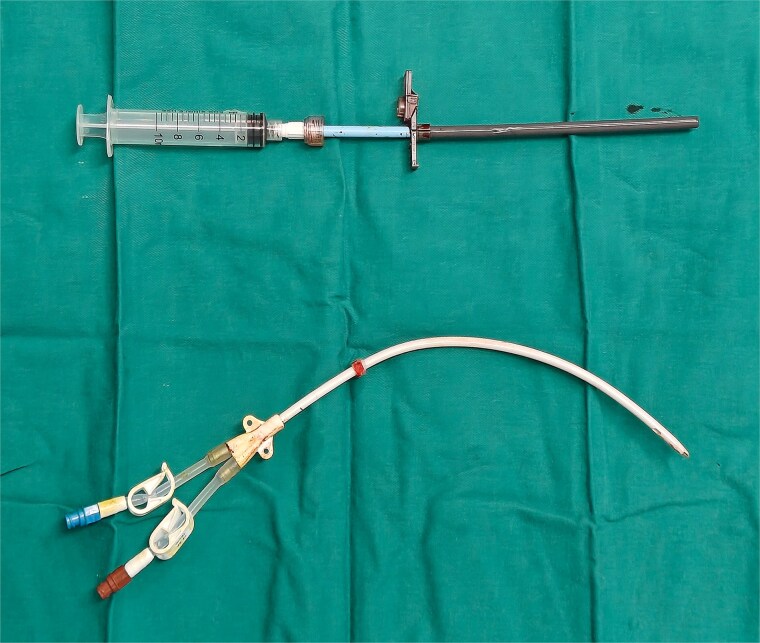
Postoperative photograph: the photograph shows the removed catheter and puncture needle.

Postoperatively, the patient remained hemodynamically stable and was transferred to the intensive care unit. No rebleeding, cardiac tamponade, or other complications were observed. A noncontrast chest CT performed on postoperative day 3 confirmed complete catheter removal, no expansion of the residual periaortic hematoma, and significant resolution of the pericardial effusion (Fig. 6). The patient was discharged on postoperative Day 7 in stable condition. At the 1-month follow-up, the patient remained asymptomatic, and no vascular complications were evident. Permanent avoidance of future puncture on the affected side was advised.

**Figure 6:**
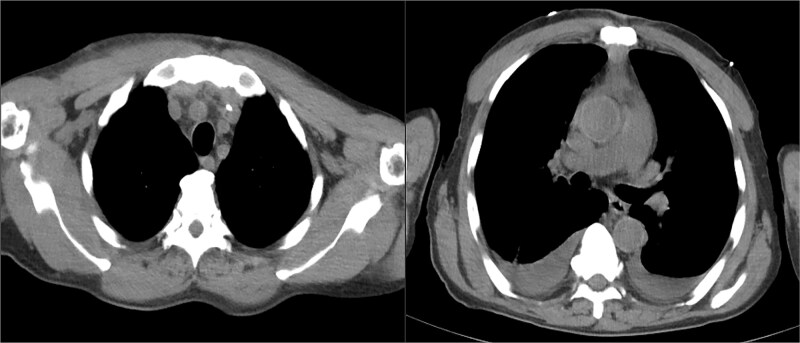
Follow-up noncontrast chest CT images: images show complete catheter removal, no expansion of the residual periaortic hematoma, and significant resolution of the pericardial effusion.

## Discussion

Inadvertent arterial cannulation during CVC for hemodialysis access is a rare but catastrophic complication, with an estimated incidence of 0.1%–2.7% across all access sites [2]. Aortic arch perforation, as observed in this case, represents an exceedingly rare and severe subset of arterial injury. Its consequences range from a localized hematoma to life-threatening hemorrhage and cardiac tamponade [6].

CTA demonstrated the catheter entering the right common carotid artery, traversing the brachiocephalic trunk, and penetrating the aortic arch, with its tip located extraluminally. This trajectory indicates a posteromedial needle angulation with excessive insertion depth, a highly suggestive of a failure to use real-time ultrasound guidance. Under ultrasound visualization, inadvertent arterial entry should be immediately recognized, allowing needle withdrawal before guidewire or dilator advancement. Progression to aortic perforation implies that the initial puncture was either unrecognized or its significance was underestimated. Lack of real-time ultrasound guidance remains the most critical modifiable factor. Real-time ultrasound guidance is the most effective preventive measure and is strongly recommended for internal jugular vein cannulation by the 2025 American Society of Echocardiography guidelines, which delineate its role in precannulation assessment, dynamic needle guidance, and postprocedural complication detection [7]. Although high-quality evidence supports the superiority of ultrasound guidance [8], its implementation in clinical practice remains suboptimal, largely due to limitations in equipment availability and provider training [9].

Once aortic arch perforation with an extraluminal catheter tip and hemopericardium is confirmed, management options are constrained by the high risk of exsanguination and cardiac tamponade. In this case, emergency open surgical exploration with standby cardiopulmonary bypass was undertaken, enabling controlled catheter removal under direct visualization with manual compression. This approach was justified by: (i) the aortic arch is a high-pressure, non-compressible mediastinal vessel, and blind removal would withdraw the sole tamponading element; (ii) the presence of hemopericardium indicates active bleeding and impending tamponade; and (iii) anatomical infeasibility of alternative approaches. Endovascular stent-grafting is viable for contained aortic injuries in stable patients, but an extraluminal catheter tip outside the pericardial cavity precludes a purely endovascular approach due to an unacceptable hemorrhage risk during removal [6, 10]. Percutaneous closure devices are primarily indicated for injuries of the subclavian and brachiocephalic arteries, and are not recommended for aortic arch injuries [6]. Conservative observation with gradual withdrawal, described for low-risk arterial injuries, is contraindicated in the presence of hemopericardium or an expanding hematoma.

Therefore, in cases of catheter-related aortic arch perforation with pericardial involvement, open surgical removal under direct vision with standby cardiopulmonary bypass represents the definitive standard of care, allowing immediate site control, pericardial drainage, and repair if needed.

## Conclusions

In conclusion, we report a rare case of catheter-related aortic arch perforation with hemopericardium following an attempted internal jugular vein cannulation. The patient underwent emergency open surgical catheter removal under direct vision with cardiopulmonary bypass standby and recovered uneventfully. While inadvertent arterial cannulation is a known complication, this case provides a novel insight: progression to aortic arch perforation with pericardial involvement constitutes a distinct entity requiring a fundamentally different approach. Blind withdrawal and endovascular strategies are contraindicated; definitive treatment mandates controlled open surgical extraction. This case underscores the need for heightened awareness and clear management protocols for this rare but catastrophic injury.

## Supplementary Material

Supplementary_Video_1_rjag521

Supplementary_Video_2_rjag521

Supplementary_Video_3_rjag521

## Data Availability

No datasets were generated or analyzed during the current study.

## References

[1] Rupp SM, Apfelbaum JL, Blitt C et al. Practice guidelines for central venous access: a report by the American Society of Anesthesiologists Task Force on central venous access. Anesthesiology 2012;116:539–73. 10.1097/ALN.0b013e31823c9569 22307320

[2] Lamperti M, Bodenham AR, Pittiruti M et al. International evidence-based recommendations on ultrasound-guided vascular access. Intensive Care Med 2012;38:1105–17. 10.1007/s00134-012-2597-x 22614241

[3] Frankel HL, Kirkpatrick AW, Elbarbary M et al. Guidelines for the appropriate use of bedside general and cardiac ultrasonography in the evaluation of critically ill patients—part I: general ultrasonography. Crit Care Med 2015;43:2479–502. 10.1097/CCM.0000000000001216 26468699

[4] Guilbert MC, Elkouri S, Bracco D et al. Arterial trauma during central venous catheter insertion: case series, review, and proposed algorithm. J Vasc Surg 2008;48:918–25. 10.1016/j.jvs.2008.05.016 18703308

[5] Wahlgren CM, Aylwin C, Davenport RA et al. Editor's choice -- European Society for Vascular Surgery (ESVS) 2025 clinical practice guidelines on the management of vascular trauma. Eur J Vasc Endovasc Surg 2025;69:1–104. 10.1016/j.ejvs.2024.12.018 39809666

[6] Dornbos DL, Nimjee SM, Smith TP. Inadvertent arterial placement of central venous catheters: systematic review and guidelines for treatment. J Vasc Interv Radiol 2019;30:1785–94. 10.1016/j.jvir.2019.05.017 31530491

[7] Vegas A, Wells B, Braum P et al. Guidelines for performing ultrasound-guided vascular cannulation: recommendations of the American Society of Echocardiography. J Am Soc Echocardiogr 2025;38:57–91. 10.1016/j.echo.2024.12.004 39909653

[8] Hind D, Calvert N, McWilliams R et al. Ultrasonic locating devices for central venous cannulation: meta-analysis. BMJ 2003;327:361. 10.1136/bmj.327.7411.361 12919984 PMC175809

[9] Zhang X, Zhang R. Real-time ultrasound-guided subclavian vein cannulation: should it be the preferred method for central venous catheterization in critically ill? Crit Care 2025;29:541. 10.1186/s13054-025-05824-7 41476201 PMC12756923

[10] Patel SJ, Venn GE, Redwood SR. Percutaneous closure of an iatrogenic puncture of the aortic arch. Cardiovasc Intervent Radiol 2003;26:407–9. 10.1007/s00270-003-0014-0 14667127

